# Morphometric Analysis and Genetic Relationship of *Rasbora* spp. in Sarawak, Malaysia

**DOI:** 10.21315/tlsr2020.31.2.3

**Published:** 2020-08-06

**Authors:** Aimi Wahidah Aminan, Leonard Lim Whye Kit, Chung Hung Hui, Badiozaman Sulaiman

**Affiliations:** 1 Faculty of Resource Science and Technology, Universiti Malaysia Sarawak, 94300 Kota Samarahan, Sarawak, Malaysia; 2 Faculty of Industrial Science and Technology, Universiti Malaysia Pahang, 26600 Pekan, Pahang, Malaysia

**Keywords:** Cryptic Diversity, *COI*, Morphometric, Molecular, *Rasbora* spp, Kepelbagaian Krip, *COI*, Morfometrik, Molekular, *Rasbora* spp

## Abstract

The genus *Rasbora* is one of the most species-rich genus among the freshwater fishes and cryptic diversity has been a major hindrance in species identification in the past four decades due to their high similarities in terms of morphology. This study aimed to investigate this issue both morphologically and molecularly. In this study, a total of 23 morphometric parameters were used to differentiate the 103 *Rasbora* fish samples harvested from different regions of Sarawak state of Malaysia via Multivariate Stepwise Discriminant Function Analysis (SDFA). Then, *cytochrome oxidase subunit I* (*COI*) gene was utilised to further distinguish 33 of these fishes, followed by sequence and phylogenetic analysis. Our results unravelled pre-anal length as strongest morphometric discriminant (100%) and that all eight *Rasbora* species tested are monophyletic except for *R. sumatrana* and *R. caudimaculata*, revealing possible cryptic *Rasbora* species. Further investigations are vital to enrich the data from this study for *Rasbora* cryptic diversity and conservation studies in future.

## Abstract (ms)

Genus *Rasbora* adalah salah satu daripada genus yang memiliki kerencaman spesies yang paling tinggi dalam kalangan ikan air tawar dan kepelbagaian krip telah menjadi penghalang utama dalam pengecaman spesies sepanjang empat dekad disebabkan persamaannya yang tinggi dari segi morfologi. Kajian ini bertujuan untuk mengkaji isu-isu morfologi dan molekular. Dalam kajian ini, 23 parameter morfometrik telah digunakan untuk membezakan 103 sampel ikan *Rasbora* yang dikutip daripada daerah berbeza di negeri Sarawak, Malaysia menggunakan *Multivariate Stepwise Discriminant Function Analysis* (SDFA). Kemudian, gen *cytochrome oxidase subunit I* (*COI*) digunakan untuk membezakan 33 jenis ikan ini, diikuti dengan analisis jujukan dan analisis filogenetik. Keputusan menunjukkan panjang pra-anal sebagai morfometrik diskriminan terkuat (100%) dan kesemua lapan spesies *Rasbora* yang diuji adalah monofiletik kecuali *R. sumatrana* and *R. caudimaculata*, dan mendedahkan spesies *Rasbora* yang mungkin krip. Penyelidikan lebih lanjut adalah sangat penting untuk memperkayakan data dari kajian ini untuk kepelbagaian dan pemuliharaan krip di masa akan datang.

**Highlights**

- Total of 23 morphometric parameters were used to differentiate the 103 *Rasbora* samples from Sarawak.
- *Cytochrome oxidase subunit I* (*COI*) gene was utilised to further distinguish these fishes through phylogenetic analysis.
- Pre-anal length identified as strongest morphometric discriminant (100%) and all eight Rasbora species tested are monophyletic except for *R. sumatrana* and *R. caudimaculata*.

## INTRODUCTION

The *Rasbora* fish are from the family of Cyprinidae and they are small to moderate in size, inhabiting the Asian region. The *Rasbora* genus is the most species-rich genus in the cyprinid Danioninae subfamily (Lumbantobing 2014). Currently, a total of 150 *Rasbora* species had been discovered where 39 species are distributed across Borneo and six are distributed across Sarawak (Fricke *et al*. 2019). There are a total of eight *Rasbora* groups identified by Brittan (1984), which are *R. trifasciata, R. argyrotaenia, R. einthovenii, R. daniconius, R. lateristriata*, *R. caudimaculata, R. sumatrana-elegans* and *R. pauciperforata* comprises of several species that are closely related according to their similar obvious features and linked evolutionarily. Taxonomically, *Rasbora* genus is known as catch all group due to insufficient unique diagnostic characteristics per species and morphological difficulty in characterisation (Muchlisin *et al*. 2012). This explains why the genus experiences cryptic diversity which this term translates as comprising more than one species that are morphologically indistinguishable and thus being characterised as a single species.

According to the International Union for Conservation of Nature (IUCN) Red List of Threatened Species, there are currently ten *Rasbora* species being categorised as “Near Threatened” and above, with two being labelled critically endangered, two labelled endangered, four labelled vulnerable and the remaining four labelled near threatened (IUCN 2019). These fishes, discovered to inhabit river streams, waterfalls and peat swamps, are deemed one of the important contributors towards the diversity of the peat swamp ecosystem (Sule *et al*. 2018). However, they are mostly threatened by residential and commercial development, natural system modifications, agriculture and aquaculture, invasive and other problematic species, genes and diseases, energy production and mining as well as pollution (IUCN 2019). For instance, *R. tawarensis* is one of the IUCN Red List’s critically endangered *Rasbora* species only found in Lake Laut Tawar, Aceh, Indonesia that is severely affected by fishing and pollution (Lumbantobing 2019). Conservation efforts such as gill nets size regulation as well as pesticide and chemical fertilisers ban have been implemented for conservation purposes (Lumbantobing 2019). In the case of the Sarawak state of Malaysia, an investigation on water quality in downstream of Bakun Dam revealed the impact of water quality on fish diversity and the *R. caudimaculata*, *R. borneensis*, *R. dusonensis* and *R. volzi* are some of the many fishes affected by the poor water quality (Liew 2016). Up to the year 2012, the Sarawak state is home to 35 endemic species (23.8% of total found in Borneo) from Cyprinidae family. At least 16 *Rasbora* species discovered to be endemic to Borneo and 14 of them are distributed across Sarawak Rajang River and Danau Sentarum National Park (Sulaiman & Mayden 2012). This calls for the urgency and needs to initiate conservation efforts to preserve *Rasbora* fishes, especially in Sarawak, as the awareness is scarce and lacks research attention. Therefore, accurate diagnostic of cryptic species complexes like that of the *Rasbora* fishes is crucial due to constantly increasing cases of natural ecosystem destruction and disturbance leading to the extinction of species. Furthermore, research focusing on putting the *Rasbora* genus into the limelight is very much in its infancy, with researches focusing on ATP-binding cassette transporter gene family in *R. sarawakensis* (Lim *et al*. 2018), whole mitogenome sequencing (Miya 2009; Ho *et al*. 2014; Zhang *et al*. 2014; Kusuma *et al*. 2017; Chung *et al*. 2020; Lim *et al*. 2019) as well as ecotoxicology (Wijeyaratne & Pathiratne 2006). Adding to that, the border security for economic activity control requires precise cryptic species determination to resolve the invasive species issues.

Morphological analysis has been a major approach in species characterisation, however this approach becomes challenging when it comes to distinguishing species complexes (group of closely related species). The challenges involved are difficulty in recognising juvenile specimens (as morphological analysis depends on life stage and species gender) as well as the overlooking of the morphologically cryptic taxa (as the analysis rely on phenotypic characteristics). This implicates the rise of issue or argument upon species discovery accuracy in future and thus complicating the identification of cryptic species. The morphological analysis, when combined with genetic analysis is powerful in distinguishing sibling species (Rius & Teske 2013). It had been used in the study of cryptic diversity of *Pacifastacus leniusculus* by Larson *et al*. (2012). The objectives of this study are to evaluate the cryptic diversity of *Rasbora* fishes from Sarawak via morphological and molecular approaches as well as to resolve their phylogenetic relationships across *Rasbora* fishes from other regions. In this study, two different approaches were used, namely morphometric analysis as well as *Cytochrome oxidase I* (*COI*) to infer the genetic relationship in order to complement the morphological identification. A total of 23 morphometric measurements were recorded emulating that of Lubantobing (2014) to determine the statistically significant differences in characteristics that are able to discriminate selected *Rasbora* species. Besides, phylogenetic analysis was conducted to resolve the relationship between the *Rasbora* fishes isolated from Sarawak and across other regions.

## MATERIALS AND METHODS

### Sample Collection

The sampling locations were listed in Table 1. These sites were randomly selected depending on its accessibility by car and boat. In order to mark the location of each sampling site, the geographical coordinates were recorded using global positioning system (GPS). The sampling activity was conducted with a permit given by Sarawak Forestry Department (NCCD.94047(Jld13)-178). Altogether, 103 *Rasbora* fishes were caught at all 12 targeted locations of Sarawak, Malaysia. Some of these were caught at slow moving drainage and also small ponds with leaves covering the water surface. Cast net, scoop net and the fish traps were used to catch the *Rasbora* fishes.

All samples were held in the portable fish bucket with oxygen supplied by the portable air pump. The samples were identified in the field whenever it is possible. The standard length and total length of each specimen was measured at the sampling site in millimetre (mm) unit. The samples were then preserved in 95% of ethanol after anesthetised with tricaine solution and brought to the laboratory for proper identification. Adult fishes are humanely sacrificed by using Tricane^TM^ as anaesthetics with permission from Universiti Malaysia Sarawak Animal Ethics Committee (reference number: UNIMAS/TNC (PI)-04.01/06-09(17)).

### Morphometric Analysis

**Figure 1:**
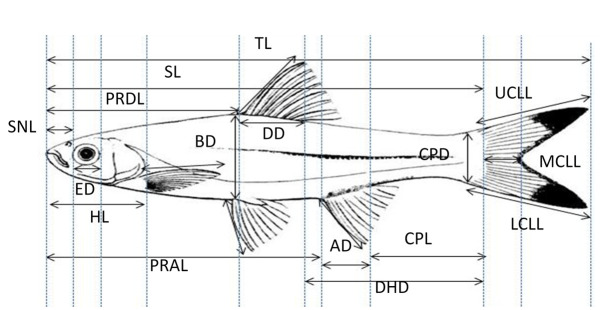
Morphometric measurements of *Rasbora* specimens. (SnL: snout length, ED: eye diameter, HL: head length, PrDL: pre-dorsal length, SL: standard length, BD: body length, PrAL: pre-anal length, TL: total length, DD: dorsal depth, CpD: caudal peduncle depth, CpL: caudal peduncle length, DHD: dorsohypural distance, LcLL: lower caudal lobe length, McLL: median caudal lobe length, UcLL: upper caudal lobe length).

A total of 23 morphometric characteristics, emulating that from Lumbantobing (2014), were measured by using vernier calliper: (a) standard length (SL), (b) total length (TL), (c) head length (HL), (d) pre-dorsal length (PrDL), (e) pre-anal length (PrAL), (f) pre-pelvic length (PrPvL), (g) dorsal depth (DD), (h) body depth (BD), (i) caudal peduncle depth (CpD), (j) caudal peduncle length (CpL), (k) dorsal fin base length (DfbL), (l) anal fin base length (AfbL), (m) pelvic length (PvL), (n) upper caudal lobe length (UcLL), (o) middle caudal lobe length (McLL), (p) lower caudal lobe length (LcLL), (q) dorsohypural distane (DHD), (r) eye diameter (ED), (s) snout length (SnL), (t) head width (HW), (u) head depth (HD), (v) inter-orbital width (IW), and (w) anal depth (AD). Fig. 1 illustrated the part of specimens used for measurement and counting. All measurements data collected were standardised to the standard length by using Covariate Adjusted Formula (AM = OM – [RC (SL–MSL)] where AM: adjusted measurement, OM: original measurement, RC: overall regression coefficient between character and standard length, SL: standard length, MSL: overall mean and standard length) by Froese *et al*. (1989) to avoid calculation bias as the specimens collected were numerous in term of size and body length. The analysis of variance (ANOVA) was conducted to determine the significant variables from all measurement characteristics used for morphometric data. The data used for ANOVA was the original measurements. The adjusted measurement data were then subjected to Multivariate Stepwise Discriminant Function Analysis (SDFA).

### Genomic DNA Extraction and PCR

A total of 33 adult fishes muscle tissues were isolated and subjected to storage in 95% ethanol. The genomic DNA was extracted using CTAB method (Chung 2018). Employing the primer pair designed by Ward et al. (2005) which are (F1 5′TCAACCAACCACAAAGACATTGGCAC 3′) and (R1 5′TAGACTTCTGGGTGGCCAAAGAATCA 3′), approximately 655 bp *COI* gene fragment was amplified via polymerase chain reaction (PCR) by using Bio-Rad T-100 Thermal Cycler. A total of 20 μL reaction volume encompassing 1.6 μL of 0.2 mM DNTPs, 2.0 μL of 1X Transtaq Buffer, 0.4 μL of 0.2 μM forward and reverse primers each, 0.2 μL of 2.5 units of TransTaq DNA polymerase and 2.0 μL genomic DNA extract was set up as follows: initial denaturation at 95°C for 2 min followed by 35 cycles of amplification 94°C for 30 s, 59.7°C for 30 s, and 72°C for 30 s, followed by 1 min final extension at 72°C. PCR products were size-separated on 1.5% agarose gel electrophoresis followed by purification and sequencing. Bi-directed sequencing was applied to obtain a full length sequence. The forward and reverse sequences retrieved after sequencing were blasted through the NCBI Nucleotide BLAST tool server for similarity searches. All trimmed sequences were then aligned using Clustal W. The interspecific and intraspecific variations were calculated to determine the variation within species and also between species using MEGA 6 programme.

### Phylogenetic Analysis

A total of 33 *COI* sequences from *Rasbora* fish in this study together with thirteen *COI* sequences of *Rasbora* from Genbank database (Table 2) were utilised and aligned using Clustal W programme. Species relationship were illustrated by constructing phylogenetic tree via MEGA 6 programme by using Maximum Likelihood (ML) method with the model test used Kimura 2 parameter. The phylogenetic tree was run for 1000 bootstraps replication in order to determine the relationship of species for genus *Rasbora*.

## RESULTS

### Morphometric Analysis

All 23 morphometric characteristics were recorded as according to Lumbantobing (2014). The ANOVA F-statistics (Table 3) for morphometric data stipulated several characteristics that were able to significantly differentiate *Rasbora* species shown by the greater F value at (*p* < 0.05). These characteristics were HL = 9.265; DD = 33.224; BD = 29.995; UcLL = 11.527; CPL = 5.679 and ED = 6.676. *R. sumatrana* recorded the highest for mean HL at 26.09 ± 2.02 mm while *R. dusonensis* recorded 21.85 ± 1.79 mm. As for DD, *R. sarawakensis* recorded the highest mean with 30.00 ± 1.97 mm while *R. einthovenii* recorded the lowest mean with 14.95 ± 4.98 mm. Descriptive statistics for the studied samples are listed in Table 3.

**Figure 2:**
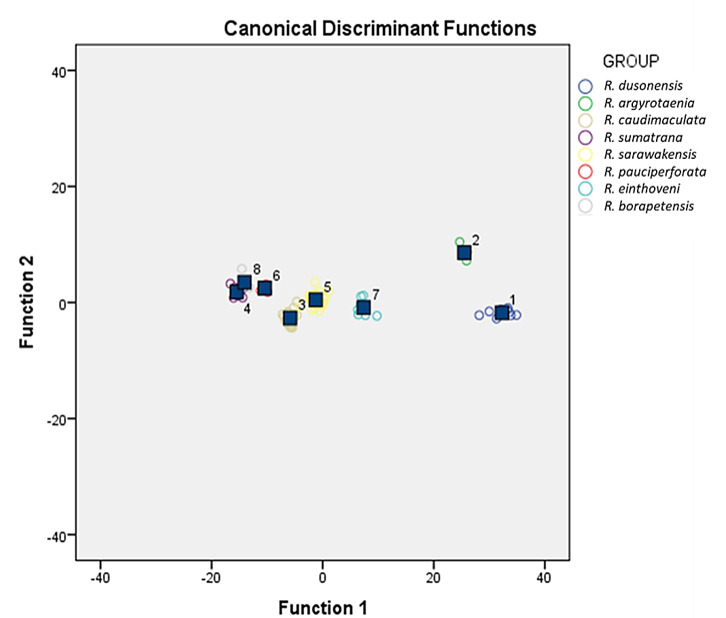
Graph of Function 1 vs. Function 2 which clearly discriminated selected *Rasbora* spp.

SDFA analysis utilised 22 morphometric measurement data results in eight groups of *Rasbora* detected as shown in Fig. 2. *R. dusonensis, R. argyrotaenia*, *R. caudimaculata, R. sarawakensis, R. einthovenii* and *R. pauciperforata* are clearly distinguished from other groups. *R. sumatrana* and *R. borapetensis* were able to distinct each other although with overlapped data points. From SDFA analysis, seven functions were determined with their variance of 93.8%, 3.3%, 1.3%, 0.8%, 0.5%, 0.2% and 0.1% respectively (Table 4). The Wilk’s lambda statistic has probability of *p* = 0.00 (Table 5) for test of Function 1 through Function 7 (Wilk’s lambda = 0.00), Function 2 through Function 7 (Wilk’s lambda = 0.004), Function 3 through Function 7 (Wilk’s Lambda = 0.030), Function 4 through Function 7 (Wilk’s lambda = 0.111), Function 5 through Function 7 (Wilk’s lambda = 0.288), Function 6 through Function 7 (Wilk’s lambda = 0.563) and Function 7 (Wilk’s lambda = 0.826). It is noted that PrAL (0.555) scores the highest followed by HL (0.536), HW (0.493), PrPvL (0.412), PrDL (0.393), BD (0.391), DHD (0.368), UcLL (0.360), HD (0.317) and PvL (0.284) when loading into SDFA according to the scoring of characteristics in Function 1 (Table 6). The contributions of these characteristics results in eight groups of predicted group *Rasbora* species with percentage of correctly classified of *R. dusonensis* (100%), *R. argyrotaenia* (100%), *R. sarawakensis* (100%), *R. borapetensis* (100%), *R. sumatrana* (100%), *R. pauciperforata* (100%), *R. caudimaculata* (100%) and *R. einthovenii* (100%) (Refer Table 7).

### Phylogenetic Relationships Inferred from Partial *COI* Gene Analysis

Sequencing of the partial *COI* gene resulted in approximately 651 bp of sequences and confirmed the absent of indels and stop codons. Pairwise sequence alignment of the partial *COI* gene encompasses 33 specimens with additional 13 sequences from of *Rasbora* species from NCBI elucidating 221 variable sites with 205 characters were parsimoniously informative. No genetic diversity detected in *R. borapetensis, R. dusonensis*, *R. pauciperforata*, with all groups constitute 0.0% interspecific variations. Genetic variation of *R. caudimaculata* ranging from 0.0% to 8.8%, *R. einthovenii* with 0.3% to 0.8% in range, *R. sarawakensis* are from 0.0% to 0.5% and *R. sumatrana* 0.0% to 3.5% display that low genetic diversity in each of *Rasbora* spp.

**Figure 3:**
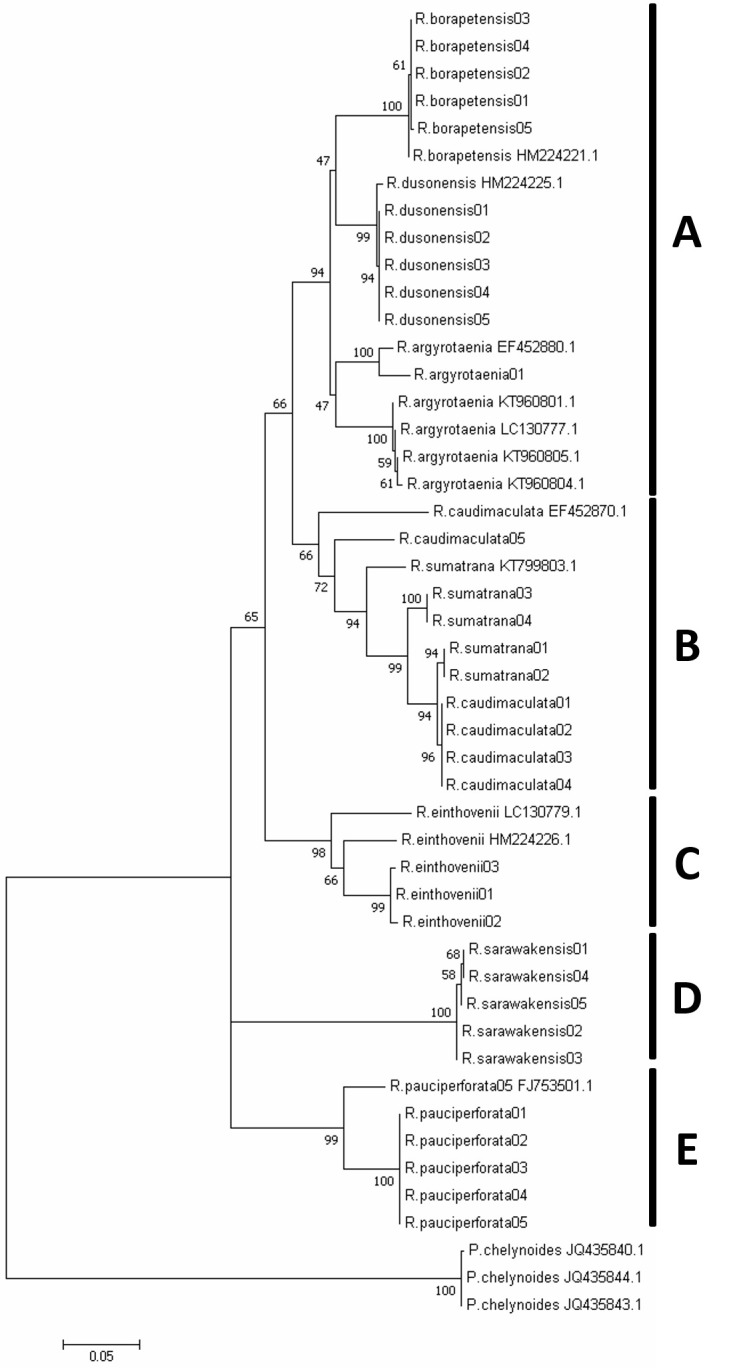
Dendogram of the phylogenetic tree constructed using maximum likelihood criterion with K2P model.

Maximum likelihood phylogenetic tree (Fig. 3) revealed that six out of eight *Rasbora* fishes (*R. borapetensis*, *R. dusonensis, R. argyrotaenia*, *R. einthovenii*, *R. pauciperforata* and *R. sarawakensis*) are monophyletic. In other words, all *Rasbora* fish species are able to form a monophyly except for *R. caudimaculata* and *R. sumatrana*. Close relationship was observed between *R. caudimaculata* and *R. sumatrana* in ML tree with bootstraps value of 100%. Low divergence between species was also discovered in this study with interspecific variations of *R. caudimaculata* and *R. sumatrana* ranging from 0.8% to 3.3%. The intraspecific variations of *R. caudimaculata* and *R. sumatrana* were 0.0% to 8.8% and 0.0% to 3.5% respectively. *R. sumatrana* and *R. caudimaculata* were found to be genetically similar and closely related despite the distinguishable morphological appearances determined via morphometric analysis.

## DISCUSSION

The discriminant analyses were accomplished to determine the functions that contributed into clustering of data to distinct groups. Essence of discriminant analysis is crucial to decide whether a characteristic is capable in differentiating an individual species from a seemingly indistinguishable sub category of organisms (Kočišová & Mišanková 2014). From SDFA analysis of morphometric data, Function 1 contributed the most in distinguishing *Rasbora* species with its variance of 93.8% and 0.00 Wilk’s lambda scores. Wilk’s lambda index described the discriminatory power by denoting 0.0 as the best discriminatory power while 1.0 as no discriminatory power (Chen *et al*. 2011).

In the Function 1, it is revealed that pre-anal length (PrAL) plays noteworthy role in distinguishing *Rasbora* species with the highest score of contribution when loading into the analysis followed by head length (HL), head width (HW), pre-pelvic length (PrPvL), pre-dorsal length (PrDL), body depth (BD), dorsohypural distance (DHD), upper-caudal lobe length (UcLL), head depth (HD) and pelvic length (PvL). The highest contribution of Function 1 was also remarked by its high cumulative percentage (93.8%) indicated that SDFA were capable of separating *Rasbora* samples into distinct species. This analysis was then concluded by the group prediction for *Rasbora* fishes with 100% correctly classified for all eight distinct species. Predicted group membership of data were based on high degree of similarity of the characteristics calculated in the analysis (Kočišová & Mišanková 2014).

The maximum likelihood phylogenetic tree constructed in this study showed six monophyletic *Rasbora* clades out of the expected eight. There are some variations and resemblances detected across the tree constructed in this study to that of Liao *et al*. (2010) that constructed phylogenetic tree based on 41 morphometric paramters. One example is that *R. enthovenii* and *R. argyrotaenia* are closely located in Liao *et al*. (2010) but on the contrary located far away in this study. In this study, *R. borapetensis* was found to reside clade in proximity to that of *R. argyrotaenia* but their positions turned out like the other end of the spectrum (far apart) in Liao *et al*. (2010). *R. enthovenii* and *R. caudimaculata* were found to locate closely in both trees compared (Liao *et al*. 2010). Comparing to the *Rasbora* phylogenetic tree constructed by Kusuma *et al*. (2016) using four different DNA barcode genes: *opsin*, *COI*, *Cytb* and *RAG1*, some similarities and differences were observed. For instances, *R. borapetensis*, *R. dusonensis* and *R. argyrotaenia* are closely located to one another in Kusuma *et al*. (2016) as well as in this study. One difference discovered is that *R. caudimaculata* is located far away from *R. sumatrana* In Kusuma *et al*. (2016) whereas that is not the case in this study.

The phylogeny of *Rasbora* genus are partially resolved in this study with some *Rasbora* species found to be non-monophyletic. In the case of *R. caudimaculata* and *R. sumatrana*, both of them shared a monophyly instead of individually, this could possibly be explained by the presence of cryptic diversity among the *Rasbora* fishes. In addition, the *R. caudimaculata* sequences mostly matches *R. sumatrana* sequences from Genbank database with very high similarity (94.0%) and the zero expected value suggests that both species might be considered as the same species. Hebert *et al*. (2003) highlighted that typical intraspecific variations are less than 3%. In this study, most of the species are correctly classified as the intraspecific divergences less than 3% except for *R. caudimaculata* and *R. sumatrana* with 8.8% and 3.5%, respectively. Given that their interspecific variations are relatively low which is 3.3% which contradict with hypothesis in determining species identification which is the intraspecific variation is significantly less than interspecific variation within barcoding sequence region (Meyer & Paulay 2005). Thus, it is suggested that there is lack of genetic differentiation at the barcode site of the sequences between these two species (Rubinoff 2006). This result supported the challenges for barcoding identified by Mallet and Walmot (2003) as there is very small variations occur in sequences between these closely related species.

In short, both *R. caudimaculata* and *R. sumatrana* are distinguishable morphologically via the SDFA analysis, but they remain unresolved when it comes to sequence and phylogenetic analysis. Similar phenomenon can be observed in *Garra imberba* and its related species (Wang *et al*. 2014) as well as *Paragonimus bangkokensis* and *P. harinasutai* (Ngoc Doanh *et al*. 2009). With this in mind, we do not exclude the possibility of the presence of cryptic species and the possibility that they are indeed the same species until further investigations, both morphologically and molecularly, are done in future.

## CONCLUSION

In this study, all 103 *Rasbora* fishes from Sarawak were classified into eight species using discriminant analyses contributed by pre-anal length characteristic as discriminatory power. Phylogenetic ML tree revealed six monophyletic *Rasbora* fish species based on *COI* sequences from this study as well as from GenBank database. *R. sumatrana* and *R. caudimaculata* were unable to form their own monophyletic cluster due to their high sequence similarities, despite them being distinguishable morphologically. Further research upon species relationship among loosely related *Rasbora* species is necessary by using two or more markers from the nuclear genome and the combination of morphological and genetic approach should be retained to provide sufficient evidences for the *Rasbora* cryptic diversity studies.

## Figures and Tables

**Table 1:** Sampling conducted in area of western of Sarawak.

Coordinate of location	Location	Total of sample
N 01 42.00′ E 109 50.20′	Gunung Gading downstream waterfall	10
1.6097° N, 110.1598° E	Sungai Rayu, Matang Wildlife Centre	5
1.7362° N, 109.8707° E	Sungai Batu, Lundu	33
1°07′07.8″ 111°31′07.8″E	Sungai Raya, Sri Aman	5
N 01 08.59′ E 110 35.02	Ranchan downstream waterfall	12
1.3070° N, 110.2947° E	Kampung Benuk	4
2°01′0″N 112°56′0″E	Ulu Kapit	10
2.7564° N, 114.0631° E	Bakun Dam	4
1.5755° N, 110.2990° E	Matang	4
1°26′52.9″N 110°25′05.6″E	Kota Samarahan	6
1.7333° N, 110.3333° E	Santubong mountain foot waterfall	5
1°27′33.7″N 110°27′18.0″E	East campus of UNIMAS	5

**Table 2:** *COI* sequences of *Rasbora* spp. retrieved from Genbank with their respective accession number and locality.

No.	*Rasbora* sequences	Accession number	Locality
1	*R. argyrotaenia*	LC130777.1	Java Island, Indonesia
2	*R. argyrotaenia*	KT960805.1	Java and Bali, Indonesia
3	*R. argyrotaenia*	KT960804.1	Java and Bali, Indonesia
4	*R. argyrotaenia*	KT960801.1	Java and Bali, Indonesia
5	*R. argyrotaenia*	EF452880.1	–
6	*R. dusonensis*	HM224225.1	Taipei, Taiwan
7	*R. sumatrana*	KT799803.1	Kota Kinabalu, Malaysia
8	*R. borapetensis*	HM224221.1	Taipei, Taiwan
9	*R. einthovenii*	LC130779.1	Java Island, Indonesia
10	*R. einthovenii*	HM224226.1	Taipei, Taiwan
11	*R. caudimaculata*	EF452870.1	–
12	*R. pauciperforata*	FJ753501.1	–
13	*P. chelynoides*	JQ435844.1	Uttar Pradesh, India
14	*P. chelynoides*	JQ435843.1	Uttar Pradesh, India
15	*P. chelynoides*	JQ435840.1	Uttar Pradesh, India

**Table 3:** Data of morphometric characteristics range with (mean±standard deviation).

Characteristics in % SL	*R.dusonensis* 86.7–110.4 mm SL N = 10	*R. argyrotaenia* 88.2–96.4 mm SL N = 4	R. *sarawakensis* 25.9–41.6 mm SL N = 27	*R. borapetensis* 16.9–23.4 mm SL N = 11	*R. sumatrana* 22.5–71.4 mm SL N = 34	*R. pauciperforata* 26.6–34.4 mm SL N = 4	*R. caudimaculata* 27.3–91.0 mm SL N = 8	*R. einthovenii* 19.4–26.4 mm SL N = 5	*F* value
**HL**	17.86–24.57 (21.85±1.79)	21.47–22.68 (22.19±0.44)	23.64–29.63 (25.87±1.40)	22.11–28.99 (25.32±1.93)	22.28–33.33 (26.09±2.02)	18.56–25.19 (22.71±2.49)	21.59–27.10 (24.31±1.84)	24.78–26.11 (25.29±0.47)	9.265^*^
**PrDL**	40.41–61.84 (50.78±5.02)	48.27–49.90 (49.32±0.66)	32.73–52.99 (48.67±3.54)	48.84–54.77 (52.13±1.82)	34.88–64.44 (51.25±4.78)	33.99–51.55 (46.32±7.19)	46.61–52.82 (50.10±2.23)	45.59–55.68 (51.05±3.43)	1.683
**PrAL**	54.73–72.17 (67.98±4.72)	65.66–66.70 (66.07±0.40)	45.17–92.03 (66.24±6.60)	63.95–74.25 (69.63±2.71)	64.29–81.33 (68.18±3.01)	64.60–66.86 (65.29±0.92)	64.23–69.72 (66.98±1.70)	65.49–69.70 (68.33±1.49)	1.073
**PrPvL**	36.63–48.91 (46.01±3.45)	45.46–47.82 (46.57±0.93)	36.06–73.96 (47.82±7.72)	41.20–66.82 (49.55±6.34)	34.22–56.89 (48.08±4.11)	45.35–51.20 (47.92±2.10)	42.28–49.67 (46.37±2.26)	36.73–42.80 (43.84±4.35)	0.764
**DD**	16.87–24.27 (22.62±2.30)	19.84–21.99 (20.80±0.81 )	25.37–33.04 (30.00±1.97)	20.18–29.27 (25.16±2.68)	17.75–28.32 (24.07±2.28)	24.74–27.07 (26.34±0.93)	20.44–25.47 (23.10±1.49)	10.18–23.20 (14.95±4.98)	33.224^*^
**BD**	19.42–26.36 (24.08±1.94)	18.55–20.54 (19.43±0.71)	26.50–31.52 (29.29±1.19)	22.49–28.80 (25.24±1.75)	20.52–29.61 (25.71±2.19)	15.41–21.80 (20.03±2.67)	20.80–24.82 (22.73±1.37)	22.57–26.44 (24.89±1.65)	29.995^*^
**CpD**	10.21–13.59 (12.35±0.87)	11.51–12.24 (11.82±0.30)	11.56–31.10 (14.40±3.42)	11.97–13.37 (12.82±0.77)	10.92–15.11 (13.64±2.03)	8.72–10.31 (9.66±0.63)	10.44–14.44 (11.71±1.20)	11.50–13.41 (12.70±0.64)	3.667
**CpL**	13.38–17.43 (16.35±2.21)	19.11–22.51 (21.07±1.24)	16.45–23.87 (20.10±1.52)	15.38–20.60 (18.32±2.45)	13.73–24.44 (19.30±2.77)	20.92–25.58 (22.48±1.83)	5.38–23.44 (16.72±5.02)	19.91–25.77 (22.92±2.25)	5.679^*^
**DfbL**	7.82–11.68 (10.24±1.15)	10.43–11.31 (10.85±0.31)	8.28–25.15 (13.40±2.83)	10.65–15.35 (12.68±1.48)	8.84–32.26 (11.77±3.74)	9.97–11.63 (11.13±1.15)	9.15–12.20 (11.04±0.93)	8.33–13.03 (10.37±1.97)	2.140
**AfbL**	6.91–11.76 (9.98±1.42)	9.64–10.61 (10.09±0.38)	10.00–14.13 (11.81±1.15)	8.54–11.73 (10.32±1.07)	7.61–13.37 (10.82±4.03)	9.48–13.91 (10.29±2.21)	10.26–13.43 (11.38±1.12)	9.47–11.86 (10.74±0.83)	0.843
**PvL**	14.65–19.84 (18.56±1.43)	16.31–18.36 (17.44±0.73)	15.07–25.93 (20.50±3.63)	13.49–26.56 (19.51±3.63)	9.78–25.33 (18.16±3.20)	15.41–19.28 (17.40±1.71 )	15.80–21.61 (18.17±1.89)	13.40–19.47 (13.57±7.08)	2.936
**PCL**	19.09–24.69 (22.81±1.50)	16.54–19.61 (18.38±1.15)	16.76–27.51 (22.89±2.62)	16.33–26.02 (22.91±4.65)	12.09–24.44 (20.86±2.90)	14.83–23.53 (17.66±3.44)	16.67–22.54 (20.03±1.57)	19.03–22.35 (21.05±1.11)	3.714
**UcLL**	20.58–33.83 (28.95±3.90)	25.14–27.28 (26.18±0.77)	27.15–37.83 (32.53±2.96)	10.71–33.70 (26.39±5.96)	19.09–33.55 (28.54±3.11)	15.04–30.07 (25.43±6.05)	27.11–33.06 (29.38±1.74)	7.58–25.22 (16.88±5.70)	11.527^*^
**McLL**	6.88–11.42 (10.32±2.94)	9.64–21.35 (13.25±4.76)	10.32–18.13 (13.71±1.61)	9.21–19.53 (14.44±2.77)	8.21–16.53 (12.09±2.06)	5.26–11.34 (9.44±2.49)	10.30–15.99 (12.30±1.77)	5.30–29.65 (17.61±8.92)	4.318
**LcLL**	24.69–37.16 (30.52±4.16)	26.48–32.74 (28.78±2.54)	22.78–36.53 (29.93±3.70)	21.14–34.78 (28.62±3.76)	7.67–43.11 (27.97±5.62)	15.04–28.18 (24.13±5.30)	27.47–33.33 (29.82±1.72)	8.71–24.74 (18.09±6.01)	4.725
**DHD**	30.70–45.33 (37.09±4.00)	34.75–42.52 (35.75±3.17)	21.88–40.12 (33.92±3.40)	28.88–38.03 (33.03±2.81)	9.07–46.67 (34.53±5.58)	33.43–37.58 (36.16±1.63)	32.60–38.21 (35.21±1.46)	31.86–42.53 (34.71±4.02)	1.109
**ED**	26.34–33.17 (29.64±2.06)	26.57–28.57 (27.59±0.72)	27.27–42.31 (35.69±3.24)	26.98–39.13 (33.57±3.66)	26.35–40.22 (33.34±3.77)	34.57–46.30 (38.22±4.79)	32.65–37.37 (35.26±1.44)	32.31–35.89 (34.47±1.27)	6.676^*^
**SnL**	3.01–6.34 (4.29±1.05)	6.22–6.93 (6.56±0.25)	2.97–6.73 (4.64±0.95)	2.80–20.74 (6.30±4.71)	2.59–28.09 (5.14±4.28)	3.59–5.15 (4.57±0.59)	2.16–5.47 (3.72±0.88)	2.65–4.64 (3.47±0.73)	0.922
**HW**	47.32–55.70 (52.59±2.52)	47.80–51.63 (49.86±1.37)	50.00–61.04 (54.14±2.90)	43.55–56.82 (50.69±3.59)	45.59–59.78 (51.68±3.67)	37.50–64.81 (49.18±9.88)	45.79–61.27 (53.97±4.30)	47.69–62.69 (54.32±4.86)	2.075
**HD**	46.09–60.56 (52.79±3.93)	48.22–52.17 (50.31±1.42)	51.04–73.08 (60.62±4.63)	48.98–70.45 (60.91±5.80)	46.67–72.91 (56.94±6.61)	51.85–74.07 (57.74±9.44)	47.66–69.12 (56.03±6.25)	59.18–67.69 (64.27±3.01)	4.313
**IW**	21.95–35.02 (28.58±3.99)	35.03–40.10 (37.57±1.82)	23.66–39.71 (30.52±3.72)	25.81–52.27 (34.14±6.95)	23.08–39.62 (30.89±4.27)	27.78–37.04 (30.70±3.72)	22.00–41.18 (31.15±5.79)	28.57–32.20 (30.09±1.25)	2.330
**AD**	5.14–19.15 (12.90±5.81 )	15.93–19.16 (17.32±1.18)	18.48–24.49 (20.67±1.49)	12.47–20.41 (16.91±2.45)	7.69–28.00 (18.57±3.56)	16.92–21.31 (19.59±1.82)	12.53–19.40 (17.61±2.06)	18.58–21.65 (16.20±8.19)	5.310

Note: N indicates the number of specimens. Characteristics: (a) standard length (SL); (b) total length (TL); (c) head length (HL); (d) pre-dorsal length (PrDL); (e) pre-anal length (PrAL); (f) pre-pelvic length (PrPvL); (g) dorsal depth (DD); (h) body depth (BD); (i) caudal peduncle depth (CpD); (j) caudal peduncle length (CpL); (k) dorsal fin base length (DfbL); (l) anal fin base length (AfbL); (m) pelvic length (PvL); (n) upper caudal lobe length (UcLL); (o) middle caudal lobe length (McLL); (p) lower caudal lobe length (LcLL); (q) dorsohypural distane (DHD); (r) eye diameter (ED); (s) snouth length (SnL); (t) head width (HW); (u) head depth (HD); (v) interorbital width (IW) and (w) anal depth (AD). Asterisk (*) indicates significant values detected by ANOVA.

**Table 4:** Eigenvalue of each function of DFA analysis of *Rasbora* spp.

Eigenvalues				

Function	Eigenvalue	% of variance	Cumulative %	Canonical correlation
1	194.763^a^	93.8	93.8	0.997
2	6.918^a^	3.3	97.1	0.935
3	2.720^a^	1.3	98.4	0.855
4	1.604^a^	0.8	99.2	0.785
5	0.954^a^	0.5	99.7	0.699
6	0.467^a^	0.2	99.9	0.564
7	0.211^a^	0.1	100.0	0.417

(a) indicates the first seven canonical discriminant functions were used in the analysis.

**Table 5:** Wilk’s lambda for each test of functions of DFA analysis of *Rasbora* spp.

Test of function(s)	Wilks’ lambda	Chi-square	df	Sig.
1 through 7	0.000	944.907	154	0.000
2 through 7	0.004	485.817	126	0.000
3 through 7	0.030	305.802	100	0.000
4 through 7	0.111	191.507	76	0.000
5 through 7	0.288	108.259	54	0.000
6 through 7	0.563	49.962	34	0.038
7	0.826	16.637	16	0.409

**Table 6:** Structure matrix of ordered correlation between measurement characteristics with functions for morphometric characteristics.

Variables	Function						

	1	2	3	4	5	6	7
PrAL	0.555^*^	0.124	−0.242	−0.025	−0.165	0.053	0.015
HL	0.536^*^	0.055	0.179	−0.189	0.056	−0.073	0.101
HW	0.493^*^	−0.087	0.144	−0.315	0.073	0.165	0.181
PrPvL	0.412^*^	0.097	−0.038	0.052	−0.189	−0.030	0.109
PrDL	0.393^*^	0.098	−0.178	−0.086	−0.097	−0.016	0.111
BD	0.391^*^	−0.308	−0.176	0.049	0.355	−0.052	−0.130
DHD	0.368^*^	0.136	−0.155	0.076	−0.165	0.193	−0.102
UcLL	0.360^*^	−0.187	0.031	0.187	−0.258	0.031	0.344
HD	0.317^*^	−0.072	0.122	−0.173	0.136	0.091	0.009
PvL	0.284^*^	−0.044	−0.127	0.171	−0.053	0.177	0.132
PCL	0.371	−0.151	−0.414^*^	0.074	0.048	0.052	0.105
AD	0.123	0.041	0.0263^*^	0.076	−0.067	0.165	0.052
DD	0.349	−0.265	0.106	0.489^*^	−0.232	0.008	0.064
CpL	0.229	0.230	0.142	0.378^*^	0.206	0.315	−0.369
ED	0.338	−0.154	0.286	−0.354	−0.378^*^	0.152	−0.366
CPD	0.212	−0.039	0.023	0.022	0.279^*^	−0.113	0.103
McLL	0.109	0.083	0.049	0.045	0.058	0.388^*^	0.340
AfbL	0.193	−0.016	0.053	−0.036	−0.180	0.307^*^	0.165
DfbL	0.108	0.141	−0.060	−0.164	−0.119	−0.284^*^	0.017
LcLL	0.275	−0.038	−0.083	0.127	−0.218	0.046	0.446^*^
SNL	0.055	0.126	0.137	0.089	0.014	−0.052	0.301^*^
IW	0.212	0.224	0.215	0.074	0.042	0.107	0.241^*^

*Note*: Asterisk (*) indicates the highest contribution of the measurement characteristics to the respective functions.

**Table 7:** Classification results of *Rasbora* species in SDFA using morphometric data.

Group	Predicted group membership original counts								Total number of samples	% of correctly classified

	RD	RA	RS	RB	RM	RP	RC	RE		
RD	10	0	0	0	0	0	0	0	10	100.0
RA	0	4	0	0	0	0	0	0	4	100.0
RS	0	0	27	0	0	0	0	0	27	100.0
RB	0	0	0	11	0	0	0	0	11	100.0
RM	0	0	0	0	34	0	0	0	34	100.0
RP	0	0	0	0	0	4	0	0	4	100.0
RC	0	0	0	0	0	0	8	0	8	100.0
RE	0	0	0	0	0	0	0	5	5	100.0

Note: Eight groups were detected via SDFA represented by R. dusonensis (RD), R. argyrotaenia (RA), R. sarawakensis (RS), R. borapetensis (RB), R. sumatrana (RM), R. pauciperforata (RP), R. caudimaculata (RC) and R. einthovenii (RE).
